# Tetra­ethyl­ammonium l-tartarate dihydrate

**DOI:** 10.1107/S1600536808036969

**Published:** 2008-11-13

**Authors:** Mohd Basyaruddin Abdul Rahman, Khairulazhar Jumbri, Kamaliah Sirat, Reza Kia, Hoong-Kun Fun

**Affiliations:** a Department of Chemistry, Faculty of Science, Universiti Putra Malaysia, 43400 UPM Serdang, Selangor, Malaysia; b X-ray Crystallography Unit, School of Physics, Universiti Sains Malaysia, 11800 USM, Penang, Malaysia

## Abstract

In the crystal structure of the title compound, C_8_H_20_N^+^·C_4_H_5_O_6_^−^·2H_2_O, the ions and water mol­ecules are linked *via* O—H⋯O and C—H⋯O hydrogen bonds, forming a two-dimensional network parallel to (001).

## Related literature

For hydrogen-bond motifs, see: Bernstein *et al.* (1995 ▶). For related structures, see: Allen *et al.* (2006 ▶); Jiang *et al.* (2008 ▶); Mei *et al.* (2002 ▶).
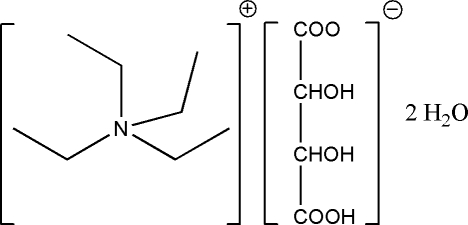


## Experimental

### 

#### Crystal data

C_8_H_20_N^+^·C_4_H_5_O_6_^−^·2H_2_O
*M* *_r_* = 315.36
Monoclinic, 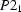

*a* = 7.4074 (1) Å
*b* = 13.8989 (2) Å
*c* = 8.0546 (1) Å
β = 106.553 (1)°
*V* = 794.89 (2) Å^3^
*Z* = 2
Mo *K*α radiation
μ = 0.11 mm^−1^
*T* = 100.0 (1) K
0.47 × 0.45 × 0.17 mm

#### Data collection

Bruker SMART APEXII CCD area-detector diffractometer
Absorption correction: multi-scan (*SADABS*; Bruker, 2005 ▶) *T*_min_ = 0.861, *T*_max_ = 0.981
10518 measured reflections
3579 independent reflections
3240 reflections with *I* > 2σ(*I*)
*R*_int_ = 0.031

#### Refinement

*R*[*F* ^2^ > 2σ(*F* ^2^)] = 0.037
*wR*(*F* ^2^) = 0.092
*S* = 1.05
3579 reflections
218 parameters
1 restraint
H atoms treated by a mixture of independent and constrained refinement
Δρ_max_ = 0.29 e Å^−3^
Δρ_min_ = −0.23 e Å^−3^

### 

Data collection: *APEX2* (Bruker, 2005 ▶); cell refinement: *SAINT* (Bruker, 2005 ▶); data reduction: *SAINT*; program(s) used to solve structure: *SHELXTL* (Sheldrick, 2008 ▶); program(s) used to refine structure: *SHELXTL*; molecular graphics: *SHELXTL*; software used to prepare material for publication: *SHELXTL* and *PLATON* (Spek, 2003 ▶).

## Supplementary Material

Crystal structure: contains datablocks global, I. DOI: 10.1107/S1600536808036969/ci2709sup1.cif

Structure factors: contains datablocks I. DOI: 10.1107/S1600536808036969/ci2709Isup2.hkl

Additional supplementary materials:  crystallographic information; 3D view; checkCIF report

## Figures and Tables

**Table 1:** Hydrogen-bond geometry (Å, °)

*D*—H⋯*A*	*D*—H	H⋯*A*	*D*⋯*A*	*D*—H⋯*A*
O2—H1*O*2⋯O5^i^	1.00 (2)	1.52 (2)	2.5108 (13)	173 (2)
O3—H1*O*3⋯O1*W*^ii^	0.91 (2)	1.85 (2)	2.7191 (14)	162 (2)
O4—H1*O*4⋯O2*W*^iii^	0.84 (2)	2.18 (2)	2.9780 (16)	160 (2)
O1*W*—H1*W*1⋯O2^iv^	0.82 (2)	2.56 (2)	3.0668 (14)	122 (2)
O1*W*—H1*W*1⋯O2*W*^v^	0.82 (2)	2.57 (2)	3.2155 (16)	137 (2)
O1*W*—H2*W*1⋯O6^iii^	0.88 (3)	2.00 (3)	2.8672 (15)	171 (2)
O2*W*—H2*W*2⋯O1^vi^	0.84 (2)	2.40 (2)	3.0082 (14)	129 (2)
C5—H5*A*⋯O3^vii^	0.97	2.56	3.4344 (15)	151
C8—H8*B*⋯O4	0.96	2.38	3.3447 (16)	178
C10—H10*B*⋯O3^vii^	0.96	2.47	3.4195 (16)	168
C11—H11*A*⋯O4	0.97	2.50	3.2693 (15)	136

Symmetry codes: (i) 
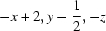
; (ii) 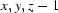; (iii) 
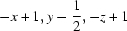
; (iv) 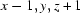; (v) 
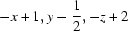
; (vi) 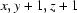; (vii) 
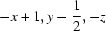
.

**Fig. 1.**
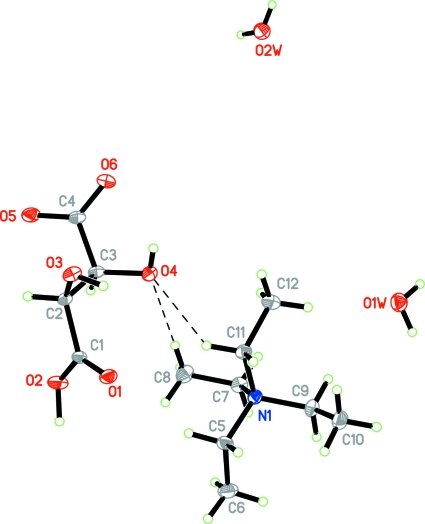
The molecular structure of the title compound, showing 50% probability displacement ellipsoids and the atomic numbering. Hydrogen bonds are shown as dashed lines.

**Fig. 2.**
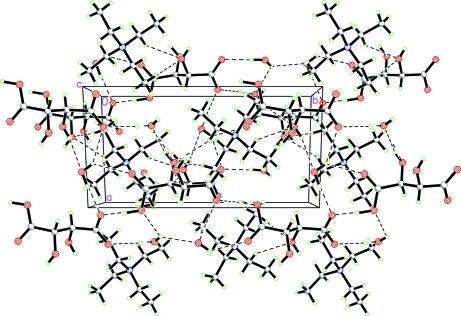
The crystal packing of the title compound, viewd down the c axis. Hydrogen bonds are shown as dashed lines.

## supplementary crystallographic information

### Comment

The crystal structures of chiral complexes of the plant acid (L-tartaric acid) with tetraethylammonium have been investigated in our laboratory in order to understand the nature of intramolecular and intermolecular interactions. The title compound was obtained by neutralization method at room temperature. Some other related compounds containing the same cation have been previously reported (Jiang *et al.*, 2008; Allen *et al.*, 2006). The crystal structure of bis(tetraethylammonium) tartrate bis(thiourea) dihydrate has also been reported (Mei *et al.*, 2002).

The asymmetric unit of the title compound (Fig. 1) contains a tartarate anion, a tetraethylammonium cation and two water molecules of crystallization. Two intermolecular C—H···O hydrogen bonds involving O4 as a bifurcated acceptor link anion and cation in the asymmetric unit to form a seven-membered ring, with *R*^1^_2_(7) ring motif (Bernstein *et al.*, 1995). In the crystal structure, the ionic units and water molecules are linked *via* O—H···O and C—H···O hydrogen bonds (Table 1) forming a two-dimensional network parallel to the (001) [Fig. 2].

### Experimental

L-Tartaric acid (7.504 g, 0.05 mol) was first dissolved in 20 ml of distilled water in a 50 ml beaker. An aqueous solution (20% in water) of tetraethylammonium hydroxide (36.59 ml, 0.05 mol) was added slowly into an aqueous solution of L-tartaric acid and the mixture was stirred with a magnetic stirrer for 2 h at room temperature. A white solid product was obtained after being dried at 343 K under vacuum for 2 d. The product was dissolved in methanol and then covered by aluminium foil to allow slow evaporation at room temperature. Clear crystalline solid was obtained after 3 d. Decomposition temperature range (471.35–472.6 K). Analysis calculated (%): C 51.60, H 9.02, N 5.01%; found: C 50.53, H 9.09, N 3.28%.

### Refinement

O-bound H atoms were located in a difference Fourier map and refined freely [O—H = 0.83 (3)–1.01 (3) Å]. C-bound H atoms were positioned geometrically [C—H = 0.93–0.98 Å] and refined as a riding model, with *U*_iso_(H) = 1.2–1.5*U*_eq_(C). A rotating group model was used for the methyl groups. In the absence of significant anomalous dispersion effects, Friedel pairs were merged before the final refinement.

### Crystal data

**Table:**

C_8_H_20_N^+^·C_4_H_5_O_6_^–^·2H_2_O	*F*_000_ = 344
*M**_r_* = 315.36	*D*_x_ = 1.318 Mg m^−^^3^
Monoclinic, *P*2_1_	Mo *K*α radiation λ = 0.71073 Å
Hall symbol: P 2yb	Cell parameters from 4555 reflections
*a* = 7.4074 (1) Å	θ = 2.6–33.5º
*b* = 13.8989 (2) Å	µ = 0.11 mm^−^^1^
*c* = 8.0546 (1) Å	*T* = 100.0 (1) K
β = 106.553 (1)º	Block, colourless
*V* = 794.891 (19) Å^3^	0.47 × 0.45 × 0.17 mm
*Z* = 2	

### Data collection

**Table:**

Bruker SMART APEXII CCD area-detector diffractometer	3579 independent reflections
Radiation source: fine-focus sealed tube	3240 reflections with *I* > 2σ(*I*)
Monochromator: graphite	*R*_int_ = 0.031
*T* = 100.0(1) K	θ_max_ = 35.0º
φ and ω scans	θ_min_ = 2.6º
Absorption correction: multi-scan(SADABS; Bruker, 2005)	*h* = −9→11
*T*_min_ = 0.861, *T*_max_ = 0.981	*k* = −22→17
10518 measured reflections	*l* = −12→12

### Refinement

**Table:**

Refinement on *F*^2^	Secondary atom site location: difference Fourier map
Least-squares matrix: full	Hydrogen site location: inferred from neighbouring sites
*R*[*F*^2^ > 2σ(*F*^2^)] = 0.037	H atoms treated by a mixture of independent and constrained refinement
*wR*(*F*^2^) = 0.092	*w* = 1/[σ^2^(*F*_o_^2^) + (0.0494*P*)^2^ + 0.0426*P*] where *P* = (*F*_o_^2^ + 2*F*_c_^2^)/3
*S* = 1.05	(Δ/σ)_max_ = 0.001
3579 reflections	Δρ_max_ = 0.29 e Å^−^^3^
218 parameters	Δρ_min_ = −0.23 e Å^−^^3^
1 restraint	Extinction correction: none
Primary atom site location: structure-invariant direct methods	

### Special details

**Table:**

Experimental. The low-temperature data was collected with the Oxford Cyrosystem Cobra low-temperature attachment.
Geometry. All e.s.d.'s (except the e.s.d. in the dihedral angle between two l.s. planes) are estimated using the full covariance matrix. The cell e.s.d.'s are taken into account individually in the estimation of e.s.d.'s in distances, angles and torsion angles; correlations between e.s.d.'s in cell parameters are only used when they are defined by crystal symmetry. An approximate (isotropic) treatment of cell e.s.d.'s is used for estimating e.s.d.'s involving l.s. planes.
Refinement. Refinement of *F*^2^ against ALL reflections. The weighted *R*-factor *wR* and goodness of fit *S* are based on *F*^2^, conventional *R*-factors *R* are based on *F*, with *F* set to zero for negative *F*^2^. The threshold expression of *F*^2^ > σ(*F*^2^) is used only for calculating *R*-factors(gt) *etc*. and is not relevant to the choice of reflections for refinement. *R*-factors based on *F*^2^ are statistically about twice as large as those based on *F*, and *R*- factors based on ALL data will be even larger.

### Fractional atomic coordinates and isotropic or equivalent isotropic displacement parameters (Å^2^)

**Table:**

	*x*	*y*	*z*	*U*_iso_*/*U*_eq_	
O1	0.71436 (14)	0.19227 (7)	−0.03566 (13)	0.01818 (18)	
O2	1.02013 (14)	0.23261 (7)	0.04449 (14)	0.0201 (2)	
O3	0.61932 (14)	0.36818 (7)	−0.18764 (12)	0.01677 (18)	
O4	0.68458 (16)	0.39108 (7)	0.17930 (13)	0.0190 (2)	
C1	0.83853 (18)	0.25107 (9)	−0.01824 (15)	0.0138 (2)	
C2	0.79871 (18)	0.35668 (9)	−0.06869 (15)	0.0134 (2)	
H2A	0.8929	0.3785	−0.1246	0.016*	
C3	0.81928 (18)	0.41837 (9)	0.09447 (16)	0.0144 (2)	
H3A	0.9446	0.4064	0.1739	0.017*	
C4	0.80699 (19)	0.52553 (9)	0.04614 (16)	0.0156 (2)	
N1	0.61784 (16)	0.13577 (8)	0.44527 (13)	0.01415 (19)	
C5	0.68066 (19)	0.05680 (10)	0.34333 (17)	0.0169 (2)	
H5A	0.5698	0.0232	0.2745	0.020*	
H5B	0.7406	0.0866	0.2638	0.020*	
C6	0.8153 (2)	−0.01643 (11)	0.4516 (2)	0.0249 (3)	
H6A	0.8474	−0.0632	0.3769	0.037*	
H6B	0.7563	−0.0481	0.5285	0.037*	
H6C	0.9275	0.0156	0.5181	0.037*	
C7	0.78506 (19)	0.18803 (10)	0.56438 (16)	0.0174 (2)	
H7A	0.7381	0.2382	0.6249	0.021*	
H7B	0.8550	0.1428	0.6507	0.021*	
C8	0.9195 (2)	0.23319 (12)	0.47596 (19)	0.0227 (3)	
H8A	1.0203	0.2644	0.5609	0.034*	
H8B	0.8532	0.2797	0.3926	0.034*	
H8C	0.9703	0.1841	0.4183	0.034*	
C9	0.50734 (19)	0.09482 (11)	0.56176 (16)	0.0186 (2)	
H9A	0.5880	0.0505	0.6428	0.022*	
H9B	0.4763	0.1471	0.6286	0.022*	
C10	0.3270 (2)	0.04279 (11)	0.46895 (19)	0.0218 (3)	
H10A	0.2674	0.0194	0.5523	0.033*	
H10B	0.3558	−0.0104	0.4047	0.033*	
H10C	0.2437	0.0864	0.3910	0.033*	
C11	0.49727 (18)	0.20335 (10)	0.31067 (15)	0.0157 (2)	
H11A	0.5737	0.2288	0.2411	0.019*	
H11B	0.3959	0.1665	0.2344	0.019*	
C12	0.4121 (2)	0.28706 (11)	0.38285 (18)	0.0205 (3)	
H12A	0.3389	0.3259	0.2891	0.031*	
H12B	0.5110	0.3253	0.4562	0.031*	
H12C	0.3325	0.2630	0.4489	0.031*	
O1W	0.30848 (16)	0.27561 (10)	0.84652 (15)	0.0243 (2)	
O2W	0.69834 (18)	0.97603 (9)	0.95574 (16)	0.0252 (2)	
O5	0.93673 (14)	0.55475 (7)	−0.01728 (15)	0.0218 (2)	
O6	0.67739 (15)	0.57466 (8)	0.07043 (14)	0.0226 (2)	
H1O2	1.043 (4)	0.161 (2)	0.045 (3)	0.045 (7)*	
H1O3	0.534 (3)	0.3304 (19)	−0.159 (3)	0.036 (6)*	
H1O4	0.590 (3)	0.422 (2)	0.122 (3)	0.033 (6)*	
H1W1	0.260 (4)	0.306 (2)	0.911 (4)	0.055 (8)*	
H2W1	0.316 (4)	0.216 (2)	0.884 (3)	0.043 (7)*	
H1W2	0.818 (4)	0.9985 (19)	0.980 (3)	0.037 (6)*	
H2W2	0.630 (4)	1.025 (2)	0.962 (3)	0.039 (6)*	

### Atomic displacement parameters (Å^2^)

**Table:**

	*U*^11^	*U*^22^	*U*^33^	*U*^12^	*U*^13^	*U*^23^
O1	0.0174 (4)	0.0115 (4)	0.0261 (4)	−0.0015 (3)	0.0070 (4)	0.0010 (4)
O2	0.0150 (4)	0.0121 (4)	0.0307 (5)	0.0009 (4)	0.0027 (4)	−0.0024 (4)
O3	0.0174 (4)	0.0135 (4)	0.0167 (4)	−0.0013 (3)	0.0006 (3)	0.0020 (3)
O4	0.0242 (5)	0.0155 (4)	0.0191 (4)	0.0013 (4)	0.0091 (4)	0.0022 (3)
C1	0.0164 (5)	0.0107 (5)	0.0146 (4)	0.0011 (4)	0.0050 (4)	−0.0005 (4)
C2	0.0151 (5)	0.0095 (5)	0.0156 (4)	−0.0007 (4)	0.0040 (4)	0.0005 (4)
C3	0.0161 (5)	0.0096 (5)	0.0162 (5)	0.0001 (4)	0.0024 (4)	−0.0005 (4)
C4	0.0164 (5)	0.0104 (5)	0.0180 (5)	−0.0001 (4)	0.0013 (4)	−0.0010 (4)
N1	0.0153 (5)	0.0138 (5)	0.0131 (4)	−0.0007 (4)	0.0037 (3)	0.0003 (3)
C5	0.0185 (5)	0.0134 (5)	0.0191 (5)	−0.0001 (5)	0.0057 (4)	−0.0026 (4)
C6	0.0232 (7)	0.0160 (6)	0.0349 (7)	0.0027 (5)	0.0071 (6)	0.0032 (5)
C7	0.0168 (5)	0.0175 (6)	0.0156 (5)	−0.0016 (5)	0.0007 (4)	−0.0013 (4)
C8	0.0191 (6)	0.0217 (7)	0.0256 (6)	−0.0052 (5)	0.0036 (5)	0.0012 (5)
C9	0.0200 (6)	0.0209 (6)	0.0162 (5)	−0.0018 (5)	0.0073 (4)	0.0025 (4)
C10	0.0196 (6)	0.0221 (7)	0.0254 (6)	−0.0029 (5)	0.0089 (5)	0.0024 (5)
C11	0.0171 (5)	0.0147 (5)	0.0140 (4)	0.0012 (4)	0.0024 (4)	0.0012 (4)
C12	0.0213 (6)	0.0161 (6)	0.0235 (6)	0.0025 (5)	0.0055 (5)	−0.0020 (5)
O1W	0.0200 (5)	0.0272 (6)	0.0262 (5)	−0.0003 (4)	0.0076 (4)	0.0019 (4)
O2W	0.0250 (6)	0.0191 (5)	0.0321 (5)	−0.0048 (4)	0.0094 (4)	−0.0044 (4)
O5	0.0187 (4)	0.0118 (4)	0.0361 (5)	−0.0022 (4)	0.0096 (4)	0.0006 (4)
O6	0.0245 (5)	0.0164 (5)	0.0286 (5)	0.0071 (4)	0.0102 (4)	0.0021 (4)

### Geometric parameters (Å, °)

**Table:**

O1—C1	1.2084 (16)	C6—H6C	0.96
O2—C1	1.3203 (15)	C7—C8	1.516 (2)
O2—H1O2	1.01 (3)	C7—H7A	0.97
O3—C2	1.4085 (16)	C7—H7B	0.97
O3—H1O3	0.90 (3)	C8—H8A	0.96
O4—C3	1.4124 (17)	C8—H8B	0.96
O4—H1O4	0.84 (3)	C8—H8C	0.96
C1—C2	1.5292 (17)	C9—C10	1.516 (2)
C2—C3	1.5400 (17)	C9—H9A	0.97
C2—H2A	0.98	C9—H9B	0.97
C3—C4	1.5356 (18)	C10—H10A	0.96
C3—H3A	0.98	C10—H10B	0.96
C4—O6	1.2382 (17)	C10—H10C	0.96
C4—O5	1.2763 (17)	C11—C12	1.516 (2)
N1—C11	1.5178 (16)	C11—H11A	0.97
N1—C7	1.5183 (17)	C11—H11B	0.97
N1—C9	1.5205 (16)	C12—H12A	0.96
N1—C5	1.5209 (17)	C12—H12B	0.96
C5—C6	1.515 (2)	C12—H12C	0.96
C5—H5A	0.97	O1W—H1W1	0.83 (3)
C5—H5B	0.97	O1W—H2W1	0.88 (3)
C6—H6A	0.96	O2W—H1W2	0.91 (3)
C6—H6B	0.96	O2W—H2W2	0.85 (3)
			
C1—O2—H1O2	110.3 (15)	C8—C7—N1	115.36 (10)
C2—O3—H1O3	110.7 (16)	C8—C7—H7A	108.4
C3—O4—H1O4	101.3 (17)	N1—C7—H7A	108.4
O1—C1—O2	124.88 (12)	C8—C7—H7B	108.4
O1—C1—C2	122.38 (11)	N1—C7—H7B	108.4
O2—C1—C2	112.74 (11)	H7A—C7—H7B	107.5
O3—C2—C1	111.28 (10)	C7—C8—H8A	109.5
O3—C2—C3	111.23 (10)	C7—C8—H8B	109.5
C1—C2—C3	110.06 (10)	H8A—C8—H8B	109.5
O3—C2—H2A	108.0	C7—C8—H8C	109.5
C1—C2—H2A	108.0	H8A—C8—H8C	109.5
C3—C2—H2A	108.0	H8B—C8—H8C	109.5
O4—C3—C4	112.59 (11)	C10—C9—N1	115.34 (10)
O4—C3—C2	110.58 (10)	C10—C9—H9A	108.4
C4—C3—C2	109.84 (10)	N1—C9—H9A	108.4
O4—C3—H3A	107.9	C10—C9—H9B	108.4
C4—C3—H3A	107.9	N1—C9—H9B	108.4
C2—C3—H3A	107.9	H9A—C9—H9B	107.5
O6—C4—O5	126.43 (13)	C9—C10—H10A	109.5
O6—C4—C3	119.17 (12)	C9—C10—H10B	109.5
O5—C4—C3	114.40 (11)	H10A—C10—H10B	109.5
C11—N1—C7	111.31 (10)	C9—C10—H10C	109.5
C11—N1—C9	111.21 (10)	H10A—C10—H10C	109.5
C7—N1—C9	105.96 (9)	H10B—C10—H10C	109.5
C11—N1—C5	105.62 (9)	C12—C11—N1	115.18 (10)
C7—N1—C5	111.49 (10)	C12—C11—H11A	108.5
C9—N1—C5	111.36 (10)	N1—C11—H11A	108.5
C6—C5—N1	115.24 (11)	C12—C11—H11B	108.5
C6—C5—H5A	108.5	N1—C11—H11B	108.5
N1—C5—H5A	108.5	H11A—C11—H11B	107.5
C6—C5—H5B	108.5	C11—C12—H12A	109.5
N1—C5—H5B	108.5	C11—C12—H12B	109.5
H5A—C5—H5B	107.5	H12A—C12—H12B	109.5
C5—C6—H6A	109.5	C11—C12—H12C	109.5
C5—C6—H6B	109.5	H12A—C12—H12C	109.5
H6A—C6—H6B	109.5	H12B—C12—H12C	109.5
C5—C6—H6C	109.5	H1W1—O1W—H2W1	106 (3)
H6A—C6—H6C	109.5	H1W2—O2W—H2W2	106 (2)
H6B—C6—H6C	109.5		
			
O1—C1—C2—O3	−20.73 (16)	C11—N1—C5—C6	175.99 (11)
O2—C1—C2—O3	159.12 (10)	C7—N1—C5—C6	54.95 (15)
O1—C1—C2—C3	103.04 (13)	C9—N1—C5—C6	−63.16 (15)
O2—C1—C2—C3	−77.11 (13)	C11—N1—C7—C8	−60.40 (15)
O3—C2—C3—O4	60.68 (13)	C9—N1—C7—C8	178.57 (12)
C1—C2—C3—O4	−63.12 (13)	C5—N1—C7—C8	57.25 (15)
O3—C2—C3—C4	−64.19 (13)	C11—N1—C9—C10	56.22 (15)
C1—C2—C3—C4	172.01 (10)	C7—N1—C9—C10	177.31 (12)
O4—C3—C4—O6	−5.84 (16)	C5—N1—C9—C10	−61.29 (15)
C2—C3—C4—O6	117.86 (13)	C7—N1—C11—C12	−60.88 (14)
O4—C3—C4—O5	174.27 (11)	C9—N1—C11—C12	57.02 (14)
C2—C3—C4—O5	−62.03 (14)	C5—N1—C11—C12	177.97 (12)

### Hydrogen-bond geometry (Å, °)

**Table:**

*D*—H···*A*	*D*—H	H···*A*	*D*···*A*	*D*—H···*A*
O2—H1O2···O5^i^	1.00 (2)	1.52 (2)	2.5108 (13)	173 (2)
O3—H1O3···O1W^ii^	0.91 (2)	1.85 (2)	2.7191 (14)	162 (2)
O4—H1O4···O2W^iii^	0.84 (2)	2.18 (2)	2.9780 (16)	160 (2)
O1W—H1W1···O2^iv^	0.82 (2)	2.56 (2)	3.0668 (14)	122 (2)
O1W—H1W1···O2W^v^	0.82 (2)	2.57 (2)	3.2155 (16)	137 (2)
O1W—H2W1···O6^iii^	0.88 (3)	2.00 (3)	2.8672 (15)	171 (2)
O2W—H2W2···O1^vi^	0.84 (2)	2.40 (2)	3.0082 (14)	129 (2)
C5—H5A···O3^vii^	0.97	2.56	3.4344 (15)	151
C8—H8B···O4	0.96	2.38	3.3447 (16)	178
C10—H10B···O3^vii^	0.96	2.47	3.4195 (16)	168
C11—H11A···O4	0.97	2.50	3.2693 (15)	136

Symmetry codes: (i) −*x*+2, *y*−1/2, −*z*; (ii) *x*, *y*, *z*−1; (iii) −*x*+1, *y*−1/2, −*z*+1; (iv) *x*−1, *y*, *z*+1; (v) −*x*+1, *y*−1/2, −*z*+2; (vi) *x*, *y*+1, *z*+1; (vii) −*x*+1, *y*−1/2, −*z*.
